# Fluoxetine Requires the Endfeet Protein Aquaporin-4 to Enhance Plasticity of Astrocyte Processes

**DOI:** 10.3389/fncel.2016.00008

**Published:** 2016-02-02

**Authors:** Barbara Di Benedetto, Victoria A. Malik, Salina Begum, Lena Jablonowski, Gabriela B. Gómez-González, Inga D. Neumann, Rainer Rupprecht

**Affiliations:** ^1^Laboratory of Neuro-Glia Pharmacology, Department of Psychiatry and Psychotherapy, University of RegensburgRegensburg, Germany; ^2^Department of Neurobiology, University of RegensburgRegensburg, Germany

**Keywords:** fluoxetine, astrocytes, glia-vasculature interface, plasticity, aquaporin-4

## Abstract

Morphological alterations in astrocytes are characteristic for post mortem brains of patients affected by major depressive disorder (MDD). Recently, a significant reduction in the coverage of blood vessels (BVs) by aquaporin-4 (AQP-4)-positive astrocyte endfeet has been shown in the prefrontal cortex (PFC) of MDD patients, suggesting that either alterations in the morphology of endfeet or in AQP-4 distribution might be responsible for the disease phenotype or constitute a consequence of its progress. Antidepressant drugs (ADs) regulate the expression of several proteins, including astrocyte-specific ones. Thus, they may target AQP-4 to induce morphological changes in astrocytes and restore their proper shape or relocate AQP-4 to endfeet. Using an animal model of depression, rats selectively bred for high anxiety-like behavior (HAB), we confirmed a reduced coverage of BVs in the adult PFC by AQP-4-immunoreactive (AQP-4-IR) astrocyte processes with respect to non-selected Wistar rats (NAB), thereby validating it for our study. A further evaluation of the morphology of astrocyte in brain slices (*ex vivo*) and *in vitro* using an antibody against the astrocyte-specific cytoskeletal protein glial fibrillary acidic protein (GFAP) revealed that HAB astrocytes extended less processes than NAB cells. Furthermore, short-term drug treatment *in vitro* with the AD fluoxetine (FLX) was sufficient to increase the plasticity of astrocyte processes, enhancing their number in NAB-derived cells and recovering their basal number in HAB-derived cells. This enhanced FLX-dependent plasticity occurred, however, only in the presence of intact AQP-4, as demonstrated by the lack of effect after the downregulation of AQP-4 with RNAi in both NAB and HAB cells. Nonetheless, a similar short-term treatment did neither modulate the coverage of BVs with AQP-4-positive astrocyte endfeet in NAB nor in HAB rats, although dosage and time of treatment were sufficient to fully recover GFAP expression in HAB brains. Thus, we suggest that longer treatment regimes may be needed to properly restore the coverage of BVs or to relocate AQP-4 to astrocyte endfeet. In conclusion, FLX requires AQP-4 to modulate the plasticity of astrocyte processes and this effect might be essential to re-establish a functional glia-vasculature interface necessary for a physiological communication between bloodstream and brain parenchyma.

## Introduction

Morphometric examinations of prefrontal cortical (PFC) regions in post mortem brains of patients with major depressive disorder (MDD) revealed alterations in the density of astrocytes, in addition to neurons (Rajkowska et al., 1999; Rajkowska and Stockmeier, 2013). Moreover, studies in animal models of stress-induced depressive-like behaviors showed an additional astrocyte pathology, with morphological differences affecting specifically this cell type (Czeh et al., 2006). Astrocytes extend processes that wrap around synapses and blood vessels (BVs), thereby regulating the functionality of neuronal circuits and of the blood-brain barrier (BBB; Koehler et al., 2009; Parpura et al., 2012). Recently, a cerebrovascular pathology has been demonstrated in older subjects suffering of MDD and patients who suffered a primary cerebrovascular disease additionally showed symptoms of mood disorders (Miguel-Hidalgo et al., 2013), thus suggesting a strong causal link between a vascular pathology and MDD. Indeed, a recent study revealed a reduced coverage of BVs by astrocytic endfeet positive for aquaporin-4 (AQP-4) in the PFC of post mortem brains from MDD patients (Rajkowska et al., 2013). AQP-4 is a plasma membrane water-transporting protein which is specifically localized to the endfeet of astrocyte processes, thereby regulating several functional properties of such processes. Its regulation of water permeability is particularly important for the modulation of astrocyte plasticity, especially for the extension and migration of astrocyte processes during neuronal activity or activity around BVs (Papadopoulos and Verkman, 2013) and to maintain the integrity of the BBB (Zhou et al., 2008). Because of their localization at the BBB, astrocytes may additionally regulate the transport of therapeutic drugs in/out of the brain through their polarized endfeet which interact with BVs (Pardridge, 1999) and mutations in proteins located to the endfeet can predict a positive or negative response to antidepressants (ADs; Uhr et al., 2008). Interestingly, a recent study has shown how ablation of the mouse gene coding for AQP-4 could disrupt responses to fluoxetine (FLX) in a chronic stress model of depressive-like behavior (Kong et al., 2009). Furthermore, aqp-4 knockout mice present cognitive deficits similar to those implicated in mood disorders (Skucas et al., 2011) and exhibit an exacerbated depressive-like behavioral response after corticosterone treatment, accompanied by impaired astrocytic functions (Kong et al., 2014).

An essential mean to understand the neurobiological underpinnings of brain pathologies is the use of animal models which reproduce the main features of human diseases and several animal models exist, which have been validated to study MDD. Among them, rats selectively bred for high anxiety-related behavior (HAB) have shown to be an appropriate tool (with face, construct and predictive validity) to study molecular risk factors which may predispose to develop depressive-like behavior (Wegener et al., 2012). Although they have been extensively characterized at behavioral and pharmacological levels, it is still unknown whether they show an astrocyte pathology such as the one described in MDD patients. Therefore, the first aim of our study was to determine if HAB rats are a proper animal model to analyze astrocyte alterations in MDD and the role of AQP-4 in depressive-like behavior. Then, we followed the hypothesis that AQP-4 might be either reduced or mislocalized in a depressive-like pathology, thus being absent from endfeet and unable to fulfill its functional role(s) around BVs. Moreover, as ADs target astrocytes (Czeh and Di Benedetto, 2013) and this targeting might be relevant to treat neuropsychiatric disorders (Di Benedetto and Rupprecht, 2013), we examined whether FLX may require AQP-4 to mediate its pharmacological modulatory effects on morphological changes in astrocytes.

## Materials and Methods

### Drugs

Fluoxetine was purchased from Sigma (Taufkirchen, Germany) and was dissolved in water for *in vitro* experiments. For *ex vivo* experiments, FLX was dissolved in phosphate buffer saline (PBS, saline).

### Animals and Drug Treatment

Adult male rats (10–12 weeks old, 280–350 g) selectively bred for HAB on Wistar background and weight-matched non-selectively bred Wistar rats (NAB) were used in this study. Breeding of HAB and NAB animals occured at the animal facilities of the University of Regensburg. Animals were housed under standard laboratory conditions in groups of four [12 hours (hrs) light: dark cycle, 22–24°C, lights on at 06:00 am, food and water *ad libitum*]. For drug treatment, FLX was dissolved on the day of the injections. Rats received an i.p. injection of either 0 (saline) or 10 mg/kg FLX twice a day for 2 days. On day 3, the animals were anesthetized with CO2 and perfused transcardially with PBS, followed by 4% paraformaldehyde (PFA, Sigma) in PBS. Brains were removed and post-fixed O/N at 4°C, cryoprotected in 25% sucrose in PBS and cut coronally at 40 μm on a cryostat. Sections were preserved in a solution with 25% ethylene glycol, 25% glycerol in PBS at -20°C until further processed for IF–IHC. Animal experiments were approved by the government of the Oberpfalz, Germany, and performed in accordance with the Guide for the Care and Use of Laboratory Animals by the National Institutes of Health, Bethesda, MD, USA.

### Cell Culture

Primary astrocytes (NAB and HAB) were isolated from cerebral cortices of postnatal day 1 (P1) rat brains. In brief, brains were isolated and cortices were dissected, cut in small pieces and digested with 0.25% trypsin containing 1 mM EDTA for 20 min (min) at 37°C with gentle shaking. The remaining tissue pieces were triturated with a fire-polished Pasteur-pipette to yield dissociated cells. Cells were centrifuged at 90 × g for 5 min, resuspended in DMEM supplemented with 10% FCS, 2 mM GlutaMAXI, penicillin (100 units/ml), streptomycin (100 μg/ml) and 0.1 mM MEM Non Essential Amino Acids (Invitrogen, Darmstadt, Germany) and seeded on poly-d-lysine-coated 75 cm^2^ flasks (Bressler et al., 1980; Allen et al., 2001). When astrocyte cultures reached 80–90% confluency, flasks were shaked to detach non-astrocytic cells, trypsinized with 0.25% trypsin containing 1 mM EDTA and seeded on 24-well plates with glass coverslips at a density of about 1 × 10^5^ cells/well.

### RNA Interference (RNAi), Cell Transfection and Drug Treatment

Short interfering RNAs (siRNAs) complementary to the rat mRNA sequence coding for AQP4 were synthesized (Dharmacon Research, Lafayette, CO). Two different siRNAs (si2, 5′-CGGACUGAUGUUACUGGUUUU-3′ and si3, 5′-UCAAUUAUACCGGAGCCAGUU-3′) were selected, which were previously published to be effective in primary rat astrocytes (Nicchia et al., 2005), together with a scrambled siRNA (scr, 5′- CCUAAGGUUAAGUCGCCCUUU-3′), which was used as negative control. All sequences were submitted to a BLAST search to verify their specific targeting of Aqp-4 mRNA (si2 and si3) and the lack of targeting of any sequence for scrRNA. Transfections were performed using Lipofectamine^TM^ 2000 (Invitrogen), following manufacturer’s instructions. In brief, 24 h before transfection, medium was changed to medium without antibiotics. Afterwards, the appropriate amount of each siRNA to reach a final working concentration of 50 nM was diluted in Opti-MEM I Reduced Serum Medium (Invitrogen), incubated at RT and then mixed with Opti-MEM I containing the appropriate amount of Lipofectamine. After a 20 min incubation time, this mixture was distributed on cells. Cells were then growing for three additional days, before changing the medium to medium without FCS 24 h before drug treatment. FLX was administrated at a final concentration of 10 μM for 48 h. Afterwards, cells were washed with PBS, fixed in PFA 4% for 20 min at room temperature (RT) and then washed again with PBS and maintained at 4°C until further processed for immunofluorescent-immunocytochemistry (IF-ICC).

### Immunofluorescent-Immunohistochemistry and Immunocytochemistry (IF-IHC and IF-ICC)

For IF-IHC in rat brains: sections from the prefrontal cortex (PFC) were selected and washed thoroughly, before permeabilization and blocking for 2 h in 0.5% Triton-X 100 + 2% Normal Goat Serum (NGS, Vector Labs, Biozol, Eching, Germany) in PBS. Afterwards, they were incubated with rabbit anti-AQP4 (1:200, Abcam, Cambridge, UK) together with mouse anti-collagen IV (1:500, Sigma) antibodies or with mouse anti-GFAP antibody (1:400, Sigma) in 0.5% TX 100 + 2% NGS in PBS O/N at 4°C. Sections were then incubated with the respective secondary antibodies, the biotinylated anti-rabbit IgG antibody (1:300, Jackson ImmunoResearch, Hamburg, Germany) with anti-mouse-Cy3 (1:250, Jackson ImmunoResearch) in 1% NGS in PBS for 2 h at RT. Afterwards, sections were washed and further incubated with avidin anti-biotin Alexa Fluor 488 (1:1000, Invitrogen) together with DAPI (1:1000, Sigma) in 1% NGS in PBS for additional 2 h at RT. Finally, all sections were then washed and mounted on slides for confocal analysis.

For IF- ICC on primary astrocytes: cells were permeabilized and blocked in 0.2% Triton-X 100 + 2% NGS in PBS for 1 h at RT. Afterwards, they were incubated O/N at 4°C with rabbit anti-AQP4 antibody (1:400, Abcam) together with mouse anti-GFAP + mouse anti-S100ß (1:400 and 1:1000, respectively, Sigma) in 1% NGS in PBS. Cells were then washed and incubated 1 h with biotinylated anti-rabbit IgG antibody (Jackson Immunoresearch, 1:300) and anti-mouse-Cy3 (1:200) in 1% NGS in PBS and afterwards 1 h with avidin anti-biotin Alexa Fluor 488 (1:1000) and DAPI (1:1000) in 1% NGS in PBS. After washing, coverslips were mounted on slides with anti-fading mounting medium (Aquapolymount, Polysciences Europe GmbH, Eppelheim, Germany) and analyzed with confocal microscopy.

### Confocal Microscopy

Confocal microscopy was performed with an Olympus confocal microscope (inverted type IX81, Olympus Europe Holding GmbH, Hamburg, Germany). For experiments in adult brains (IF-IHC), mean values for coverage were obtained from an average of 10 images (20 optical sections, 1 μm Z-step size) from at least two slices per each brain. For IF-ICC, an average of 10–25 fields from each treatment condition were acquired (1 μm Z-step size) for morphological examination. The FluoView FV1000 program (Version 2.1c; Olympus FluoView Resource Center) was used to convert the images from the proprietary file format (.oib) into tagged image file format (.tiff) files for further analysis.

### Coverage Analysis

One (.tiff) file was generated for each image which contained two color channels: a red for BVs labeled with collagen IV and a green for endfeet immunoreactive (IR) for AQP-4. The area of co-localized immunolabeling in the region of interest (ROI) was measured using a Demo version of AutoQuantX3 Program. Afterwards, to calculate the coverage index (area of co-localization/total area of vessels), the area occupied by the colocalizing pixels was divided by the total area occupied by the red pixels (representing the BVs labeled with collagen IV). This ratio was defined as the “Coverage.”

### Analysis of Processes

For morphological analysis: in a first experiment (**Figure 2C**), astrocytes were stained with GFAP and S100β to label astrocyte processes and the program NeuronJ, a plugin of ImageJ (http://www.imagescience.org/meijering/software/neuronj/) was used to count the number of processes per cell, after converting images from the confocal microscope into 8-bit color pictures to be compatible with the NeuronJ Program. In a second experiment, AQP-4 staining was used to evaluate whether observed changes in morphology correlated with changes in AQP-4 cellular content. For the analysis, we marked cell boundaries using the “lasso” tool of Photoshop CS3 and then evaluated AQP-4 signal intensity using the Histogramm function. Also for the RNAi experiment we used IF-ICC to identify and choose for the analysis cells which showed a reduction in AQP-4 signal, indicative of an efficient downregulation. Only those cells with reduced signal were further examined for the number of astrocyte processes per cell.

### Statistical Analysis

To choose the appropriate statistical test, a normality test was performed for each sample distribution. Data are plotted as means ± SEM and *N* refers to the number of independent experiments. A one-way ANOVA was used, followed by Tukey HSD *post hoc* or Dunnett’s *post hoc* tests for multiple comparisons. For comparisons between two groups, the unpaired Student’s *t*-test was used. Analyses were performed with Prism GraphPad program (GraphPad Software, Inc., La Jolla, CA, USA).

## Results

### HAB Rats are a Valid Animal Model to Study Changes in Coverage of Blood Vessels by the Endfeet Protein Aquaporin-4

**FIGURE 1:**
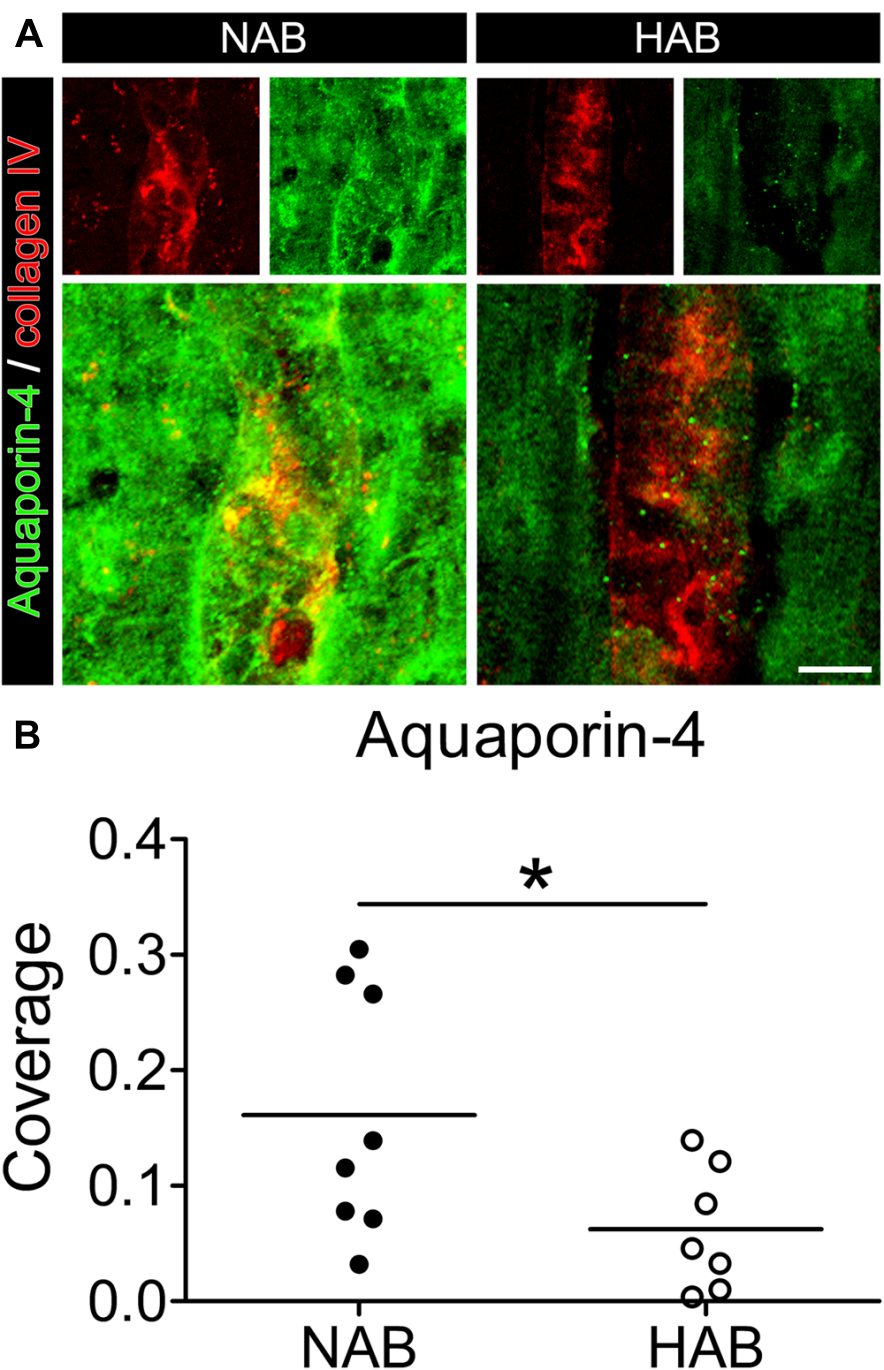
**The coverage of blood vessels (BVs) by aquaporin-4 (AQP-4)-immunoreactive astrocytic endfeet is reduced in the PFC of HAB rats with respect to NAB rats.** **(A)** Representative photomicrographs show immunofluorescent stainings for AQP-4 (in green) and collagen IV (to label BVs, in red) in NAB (left) and HAB (right) rats. **(B)** The scattered plot shows a reduction of about 60% in the coverage of BVs by AQP-4-positive endfeet in HAB rats in comparison with NAB rats [NAB (*N* = 8), HAB (*N* = 7); *t*_(1,13)_ = 2.198, ^∗^*p* < 0.05]. Scale bar = 10 μm.

First, we evaluated whether the HAB rats as the animal model used for this study, showed a reduced coverage of BVs by AQP-4-IR endfeet in the PFC, as it was shown in human post mortem brains of MDD patients (Rajkowska et al., 2013). Using immunohistochemistry to label brain slices (*ex vivo*) with collagen IV, a specific marker of BVs, together with a specific antibody against AQP-4, which is localized to the endfeet of astrocytes surrounding BVs (Papadopoulos and Verkman, 2013), we revealed that in the PFC of HAB rats the BVs showed a 60% reduction in coverage by AQP-4-IR endfeet with respect to NAB rats, similar to the finding in MDD patients [**Figure 1**; NAB, 0.16 ± 0.04, *N* = 8; HAB, 0.06 ± 0.02, *N* = 7; Student’s *t*-test, *t*_(1,13)_ = 2.198, ^∗^*p* < 0.05]. Thus, HAB rats were validated as appropriate animal model for our study.

### The Number of Astrocyte Processes is Reduced in HAB with Respect to NAB Astrocytes and Fluoxetine Treatment can Rescue this Deficit

**FIGURE 2:**
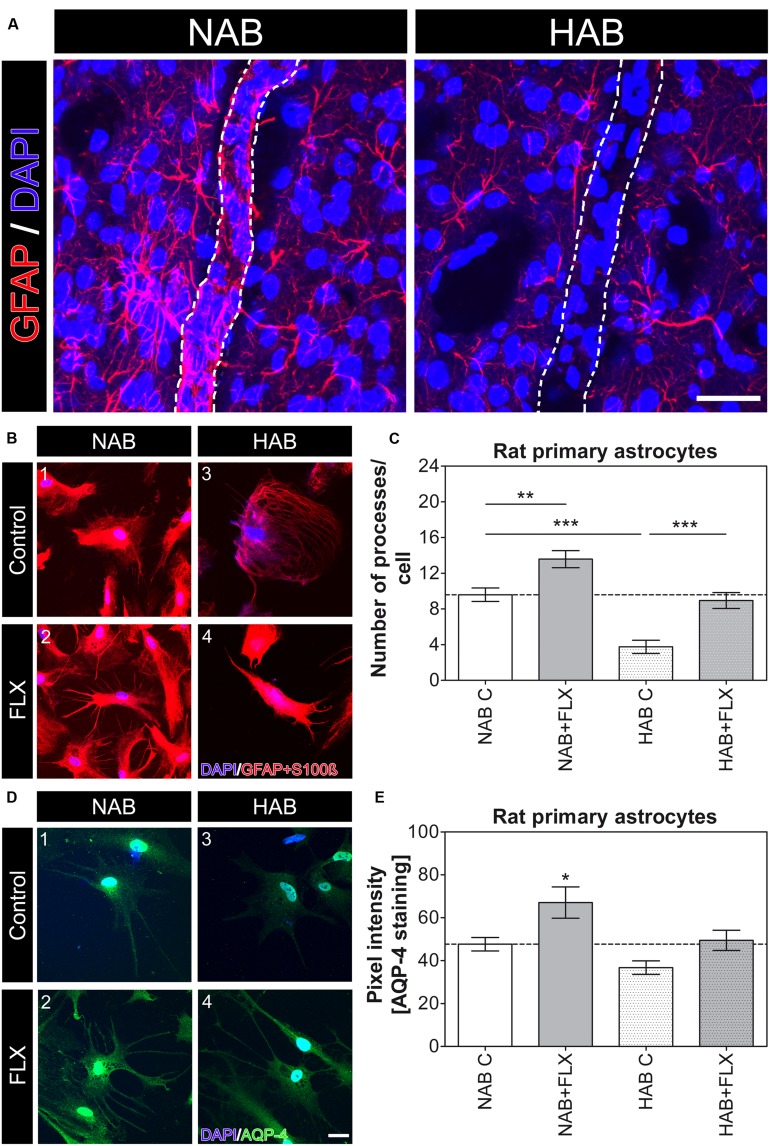
**Morphological differences in astrocytes are evident in HAB brains and HAB cells with respect to NAB and FLX can modulate astrocyte morphology *in vitro***. **(A)** Representative photomicrographs for GFAP (red) in brain slices (*ex vivo*) show a strong atrophy of astrocytes in the PFC of HAB rats. DAPI (in blue) labels nuclei of single cells to help identifying cells and BVs. Scale bar = 30 μm. **(B)** Representative photomicrographs show NAB (1 and 2) and HAB-derived (3 and 4) astrocytes before and after a 48 h FLX treatment. Cells were labelled with DAPI (blue) and the astrocyte markers GFAP and S100ß (red) to examine morphological changes. **(C)** The graph shows a reduction of about 60% in the number of astrocyte processes in HAB astrocytes in comparison with NAB cells and the effect of FLX in increasing the number of processes in both cell types [ANOVA, *F*_(1,3)_ = 23.91, ^∗∗^*p* < 0.01, ^∗∗∗^*p* < 0.001]. **(D)** Representative photomicrographs of immunofluorescent stainings show NAB (1 and 2) and HAB-derived (3 and 4) astrocytes before and after a 48 h FLX treatment. Cells were labelled with DAPI (blue) and AQP-4 (green) to measure changes in AQP-4 content per cell. **(E)** The graph shows a reduction of about 23% in the amount of AQP-4 in HAB astrocytes in comparison with NAB cells. FLX reversed AQP-4 amount to basal levels in HAB cells and significantly enhanced AQP-4 expression in NAB cells. ANOVA, *F*_(1,3)_ = 6.61, ^∗^*p* < 0.05. Scale bar = 10 μm.

To investigate whether the observed lack of coverage was dependent on a primary morphological change in astrocyte processes, causing only secondarily a loss of AQP-4-IR endfeet, or on a mislocalization of AQP-4 protein from still intact processes, we first compared *ex vivo* how GFAP, an astrocyte-specific cytoskeletal marker, was expressed in HAB and NAB brains. As previously shown (Rajkowska and Miguel-Hidalgo, 2007), we observed a dramatic reduction in the complexity of astrocytic arborization (**Figure 2A**), thereby suggesting that a main change in the morphology of astrocyte processes may underlie the reduced coverage of BVs in HAB rats. We therefore took advantage of our primary cell culture model using cortical astrocytes derived from both NAB and HAB rats to deeper examine the observed morphological differences. We thereby showed *in vitro* that HAB-derived astrocytes developed a reduced number of processes with respect to NAB-derived cells [**Figures 2B(1,3),C**, NAB C, 9.59 ± 0.75 (22 cells), HAB C, 3.75 ± 0.75 (20 cells), ANOVA, *F*_(1,3)_ = 23.91, with Tukey’s *post hoc* test, ^∗∗∗^*p* < 0.001]. As far as AQP-4 modulates extension of astrocyte processes (Saadoun et al., 2005) and a lack of AQP-4 impairs the recovery from a depressive-like phenotype upon FLX treatment in aqp-4 knockout mice (Kong et al., 2009), we then examined whether the lack of astrocyte processes in HAB cells might be linked to an AQP-4 deficiency which might thereafter disrupt responses to FLX. A short-term (48 h) FLX administration significantly increased the number of processes in NAB astrocytes, indicating such a morphological change as a FLX target. More interestingly, however, we observed that this short drug treatment was also sufficient to significantly enhance the number of astrocyte processes in HAB cells, thereby restoring the basal total number of processes per cell [**Figures 2B(2,4),C**, NAB + FLX, 13.57 ± 0.95 (21 cells), HAB + FLX, 8.94 ± 0.88 (16 cells), ANOVA with Tukey’s *post hoc* test, ^∗∗^*p* < 0.01, ^∗∗∗^*p* < 0.001]. Thus, suggesting that AQP-4 may not be functionally deficient in HAB cells to mediate FLX effect, but maybe only transcriptionally or translationally repressed. To clarify this aspect, since we in fact observed an apparent reduced AQP-4 expression in HAB brains (**Figure 1A**), we used a more quantitative method to estimate whether AQP-4 could be less expressed in HAB astrocytes with respect to NAB ones. Because commercially available antibodies against AQP-4 are better working in immunochemistry than for western blot, we performed an immunochemical staining to compare AQP-4 expression between NAB and HAB astrocytes and to evaluate how such expression was modulated upon drug administration. We thereby showed that indeed the expression of AQP-4 in HAB astrocytes was 23% less intense in average than in NAB cells, although the difference did not reach statistical significance (**Figure 2E**). More importantly, however, AQP-4 expression was significantly increased by FLX treatment in NAB astrocytes [**Figures 2D(1,2),E**, NAB C, 47.65 ± 3.17 (14 cells), NAB + FLX, 67.06 ± 7.29 (14 cells), ANOVA, *F*_(1,3)_ = 6.61, with Tukey’s *post hoc* test, ^∗^*p* < 0.05], paralleling the increased amount of astrocyte processes (**Figure 2C**). In addition, AQP-4 expression was normalized in HAB cells after FLX treatment [**Figures 2D(3,4),E**, HAB C, 36.71 ± 3.17 (14 cells), HAB + FLX, 49.46 ± 4.71 (14 cells)], corresponding to the rescued effect on numbers of processes.

### Downregulation of AQP-4 Induces a Reduction in Number of Astrocyte Processes in NAB Cells and Hampers both the Effect of FLX in NAB Cells and its Rescue Effect in HAB Cells

**FIGURE 3:**
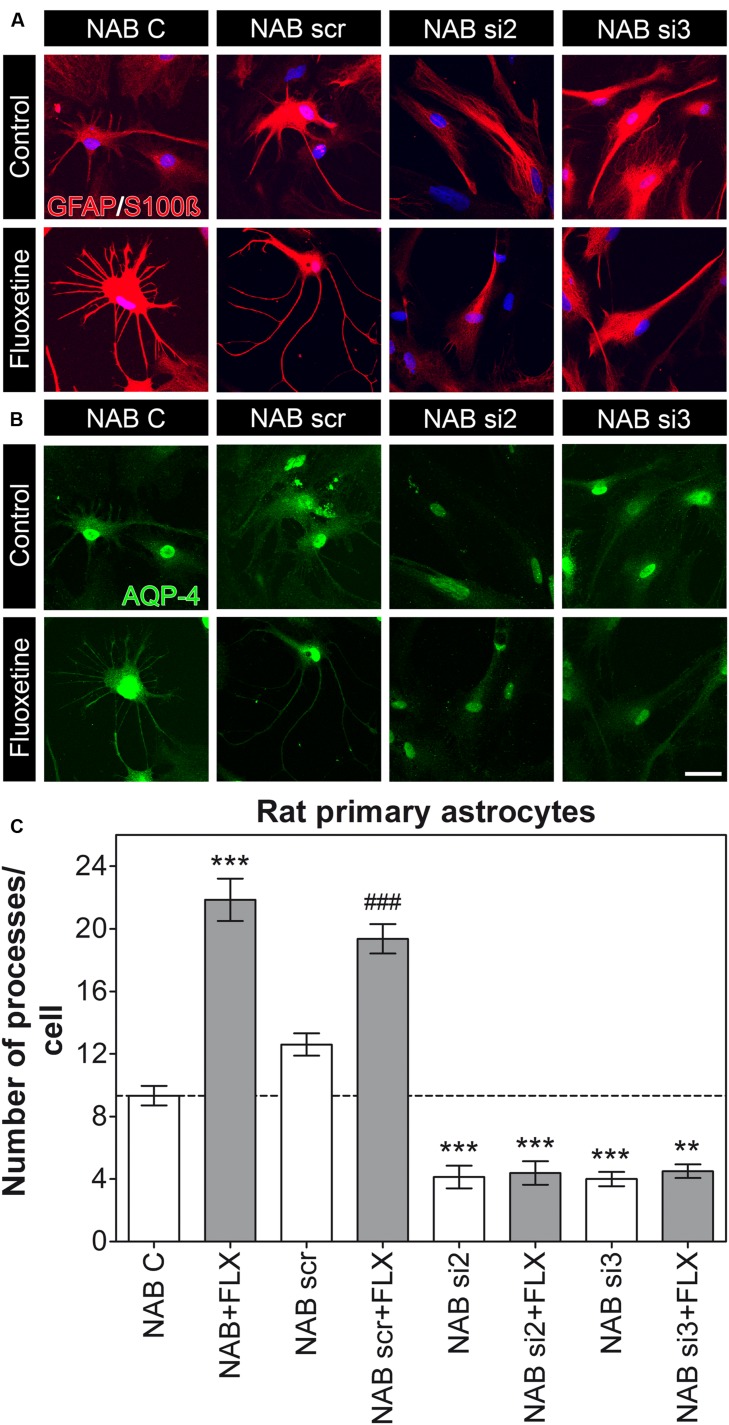
**Knockdown of AQP-4 in NAB astrocytes induces a lack of processes per cell.** **(A,B)** Photomicrographs show representative immunofluorescent stainings for GFAP/S100ß (to label astrocytes, in red) and AQP-4 (in green). **(C)** The graph shows that FLX can induce an increased amount of astrocyte processes in NAB C (control) and NAB scr (transfected with a scrambled siRNA) astrocytes, but not in cells transfected with siRNAs against the mRNA coding for AQP-4 (si2 and si3; ANOVA with Tukey HSD *post hoc* test, ^∗∗^*p* < 0.01, ^∗∗∗^*p* < 0.001 with respect to NAB C and ### *p* < 0.001 with respect to NAB scr). Scale bar = 20 μm.

To then evaluate whether AQP-4 might be responsible for the altered morphology observed in HAB cells and whether it is required for the rescue effect upon FLX treatment in these cells, we used RNAi to downregulate AQP-4 in primary astrocyte from both NAB and HAB rats. For these experiments we used siRNA sequences which already showed their AQP-4 knockdown efficacy in rat astrocytes (Nicchia et al., 2005). We thereby revealed that AQP-4 was indeed necessary for NAB astrocytes to form their processes, since its knockdown caused a reduction in the total number of processes per cell. Moreover, AQP-4 knockdown caused a loss of response to FLX treatment [**Figure 3**, NAB C, 9.33 ± 0.62 (15 cells), NAB scr, 12.6 ± 0.71 (15 cells), NAB si2, 4.13 ± 0.72 (15 cells), NAB si3, 4.00 ± 0.46 (15 cells), NAB + FLX, 21.86 ± 1.35 (14 cells), NAB scr + FLX, 19.36 ± 0.94 (14 cells), NAB si2 + FLX, 4.38 ± 0.75 (13 cells), NAB si3 + FLX, 4.5 ± 0.43 (12 cells), ANOVA, *F*_(1,7)_ = 81.21, with Tukey’s *post hoc* test, ^∗∗^*p* < 0.01, ^∗∗∗^*p* < 0.001 with respect to NAB C and ### *p* < 0.001 with respect to NAB scr]. In this experiment we could observe a higher number of astrocyte processes in response to FLX treatment than those measured in the first experiment (**Figure 2C**). However, baseline number of processes per cell were very consistent, thereby suggesting that drug effects may vary among experiments. Therefore, we focused our analysis on relative changes after FLX treatment and did not make specific claims about absolute numbers.

**FIGURE 4:**
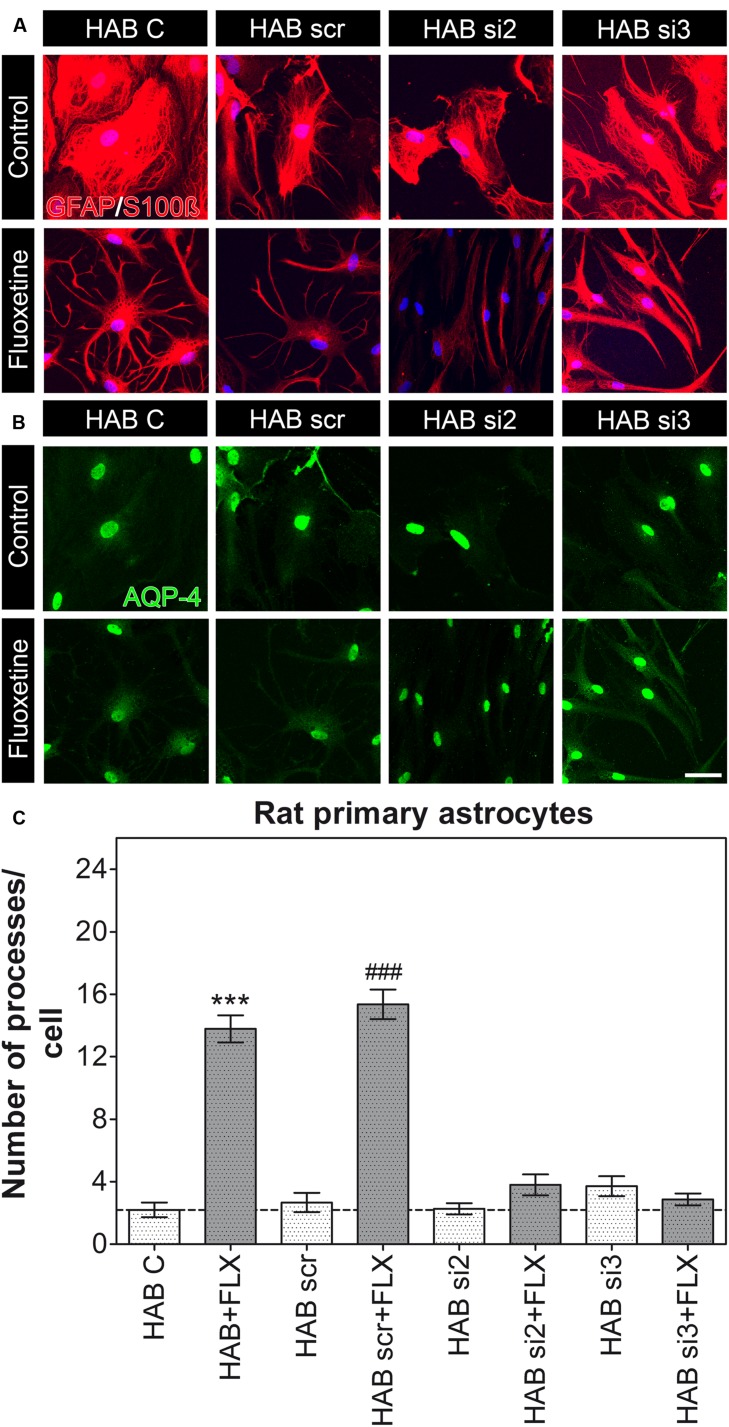
**Knockdown of AQP-4 in HAB astrocytes.** **(A,B)** Photomicrographs show representative immunofluorescent stainings for GFAP/S100ß (to label astrocytes, in red) and AQP-4 (in green). **(C)** The graph shows that FLX could still induce an increased amount of astrocyte processes in HAB C (control) and HAB scr astrocytes, thereby suggesting the presence of a still functional AQP-4 to mediate such an effect. However, cells transfected with si2 and si3 did not show the rescue effect after FLX treatment, as they remained lower than NAB (ANOVA with Tukey HSD *post hoc* test, ^∗∗∗^*p* < 0.001 with respect to HAB C and ### *p* < 0.001 with respect to HAB scr). Scale bar = 20 μm.

Furthermore, because we measured a reduced amount of AQP-4 in HAB cells, which correlated to the lowered number of processes (**Figure 2E**), we additionally performed a knockdown experiment in HAB astrocytes to verify whether such variations in AQP-4 content might affect the functional role of AQP-4 in HAB cells too. We confirmed that a partially reduced amount of AQP-4 could indeed be quantified in control HAB cells (HAB C) with respect to NAB C cells (**Supplementary Figure S1**), which might have caused the reduced amount of processes per cell in HAB astrocytes. However, a dysfunctional AQP-4 in HAB astrocytes could be ruled out because FLX was still able to restore the basal number of processes in these cells, whereas the effect of FLX was blunted in AQP-4 knockdown HAB astrocytes in which FLX could not reactivate AQP-4 expression (**Figure 2E**). Thus, further supporting the essential role of AQP-4 to mediate the growth of processes upon FLX administration [**Figure 4**, HAB C, 2.20 ± 0.47 (15 cells), HAB scr, 2.67 ± 0.61 (15 cells), HAB si2, 2.27 ± 0.36 (15 cells), HAB si3, 3.71 ± 0.64 (14 cells), HAB + FLX, 13.79 ± 0.86 (14 cells), HAB scr + FLX, 15.36 ± 0.95 (14 cells), HAB si2 + FLX, 3.80 ± 0.66 (10 cells), HAB si3 + FLX, 2.87 ± 0.38 (15 cells), ANOVA, *F*_(1,7)_ = 73.00, with Tukey’s *post hoc* test, ^∗∗∗^*p* < 0.001 with respect to HAB C and ###*p* < 0.001 with respect to HAB scr]. In this experiment we found that the siRNA si3 showed lower efficiency than si2 in downregulating AQP-4, as it was already evident in NAB cells (**Supplementary Figure S1A**). This lower efficacy of si3 gave the impression of an apparent lack of effect on AQP-4 in HAB cells (**Supplementary Figure S1B**), because in these cells the basal AQP-4 content was already quite low. However, we could still observe its functional efficacy to block the effect of FLX on astrocyte processes. This finding further supported that only in case of a genetic blockade of the drug-dependent increase of AQP-4 expression in HAB cells, which hinders the normalization of its expression (**Figure 2E**), the effect of FLX on the extension of processes was inhibited.

### Short Term FLX Treatment Modulates GFAP Expression but it is not Sufficient to Affect Coverage of BVs by Astrocytic Endfeet Neither in NAB Nor in HAB Rats

**FIGURE 5:**
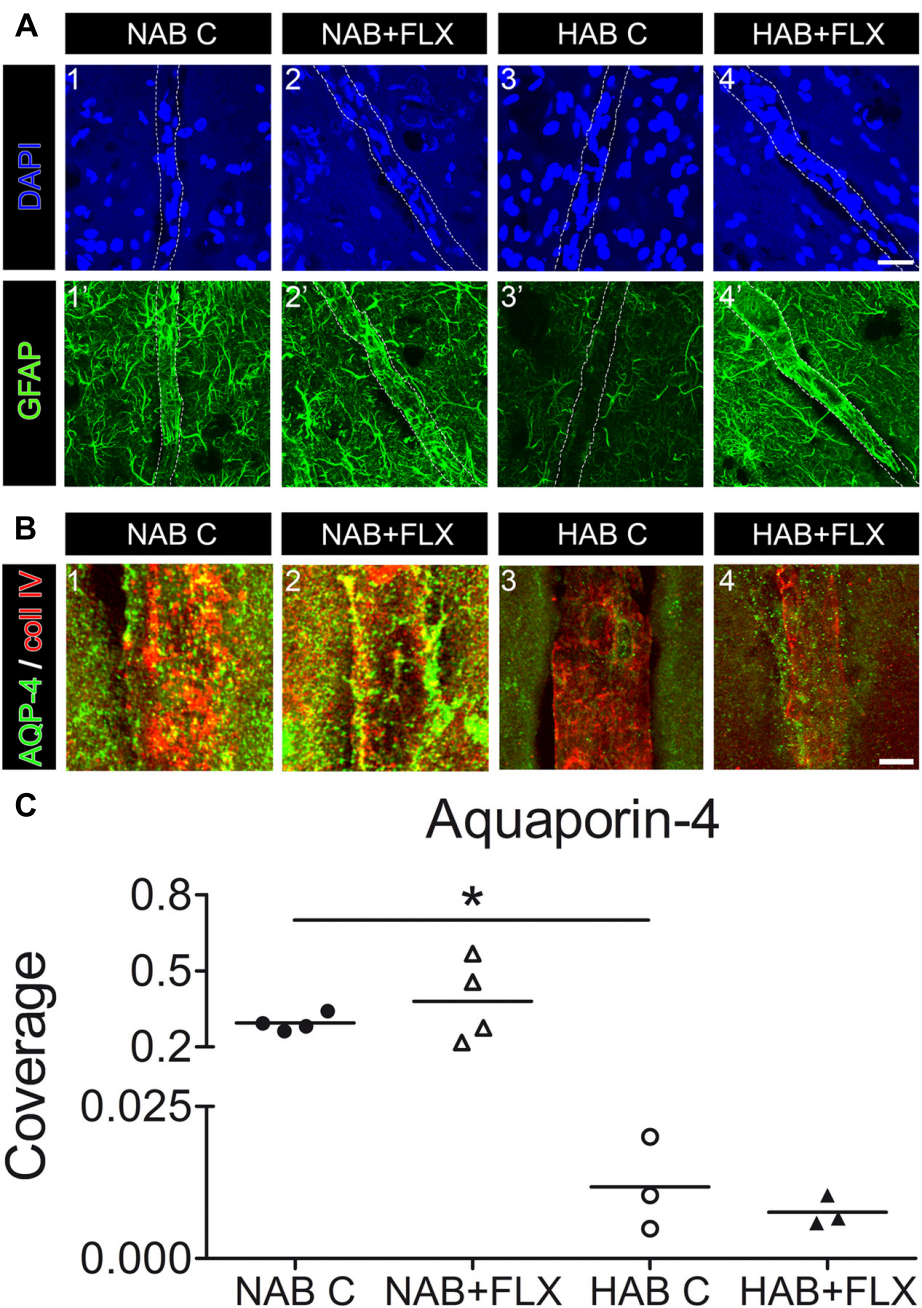
**Short-term treatment of NAB and HAB rats with FLX can reverse the reduced GFAP expression in HAB rats, but it is not sufficient to neither modulate coverage of BVs by AQP-4-IR endfeet in NAB brains nor rescue the lack of coverage in HAB brains.** **(A)** Representative photomicrographs show immunofluorescent stainings for GFAP (in green) and DAPI (in blue) in NAB (1,1’) and HAB (3,3’) brains, where it is evident the atrophy of astrocyte processes shown by the reduced GFAP staining in HAB brains (3’). Such a reduced GFAP expression could be rescued by the short-term treatment with FLX (HAB + FLX, 4,4’). Scale bar = 20 μm. **(B)** Representative photomicrographs shows that FLX did not change the coverage of BVs by AQP-4-positive endfeet neither in NAB (1,2) nor in HAB (3,4) treated rats. **(C)** The scattered plot shows that FLX did not change the coverage of BVs by AQP-4-positive endfeet neither in NAB (1,2) nor in HAB (3,4) treated rats after the short-term drug treatment [NAB, 0.294 ± 0.017, *N* = 4; NAB + FLX, 0.379 ± 0.08, *N* = 4; HAB, 0.012 ± 0.004, *N* = 3; HAB + FLX, 0.008 ± 0.001, *N* = 3; ANOVA, *F*_(1,3)_ = 15.44, with Tukey’s *post hoc* test, ^∗^*p* < 0.05]. Scale bar = 5 μm.

To verify *in vivo* whether the short-term treatment with FLX was sufficient to modulate the coverage of BVs by AQP-4-IR endfeet, we initially injected a small group of NAB and HAB rats with FLX for 48 h. Using IF-IHC on brain slices from the PFC we could show that indeed 48 h were sufficient to modulate the cytoskeletal protein GFAP, with a consequent full recover of its reduced expression in HAB brains [**Figure 5A(3’,4’)**, yet suggesting that a drug effect indeed occurred after the short-term treatment. However, we observed that FLX did not induce any significant change in the coverage of BVs by AQP4-IR endfeet in neither group of rats, although in NAB rats we found a higher degree of variability in response to FLX with respect to HAB rats [**Figures 5B(1–4),C**; NAB, 0.294 ± 0.017, *N* = 4; NAB + FLX, 0.379 ± 0.08, *N* = 4; HAB, 0.012 ± 0.004, *N* = 3; HAB + FLX, 0.008 ± 0.001, *N* = 3; ANOVA, *F*_(1,3)_ = 15.44, with Tukey’s *post hoc* test, ^∗^*p* < 0.05].

## Discussion

Several reports in the last years have suggested AQP-4 as an essential brain protein for a variety of astrocytic functions, among which water balance and K+ buffering are the mostly known (Papadopoulos and Verkman, 2013). In addition to that, more recent studies have evidenced a role of AQP-4 as mediator of AD efficacy (Kong et al., 2009) and as a “protective” factor against corticosterone-induced depressive-like phenotype accompanied by an astrocytic pathology (Kong et al., 2014), proposing AQP-4 as a novel putative molecule affected in the etiopathogenesis of MDD. In accordance with these studies, the examination of post mortem brains of MDD patients revealed a lack of coverage of BVs with AQP-4-IR endfeet of astrocytes, further supporting a role for AQP-4 in MDD (Rajkowska et al., 2013). Morphological changes in astrocytes strongly correlate with their functionality and it has been already shown that a reduction in the soma size of hippocampal astrocytes characterize the hippocampus of tree shrews after long-term psychosocial stress, which induces depressive-like behavior (Czeh et al., 2006). However, to identify risk factors which might predispose an individual to develop a depressive disorder, we would need an animal model which displays a depressive-like phenotype with a strong genetic component. We therefore decided to examine the morphology of astrocyte in HAB rats (Neumann et al., 2011; Wegener et al., 2012). Using IF-IHC we could indeed show that HAB rats show a similar reduction in the coverage of BVs by AQP-4-IR endfeet in the PFC as described in the human study by Rajkowska et al. (2013). Our results demonstrate the validity of this animal model to study the importance of an astrocyte pathology for MDD, in addition to the Wistar-Kyoto rat strain (Gosselin et al., 2009), thereby offering an alternative animal model to examine the role of astrocytes in MDD with a particular focus on glia-vasculature dysfunctions.

Furthermore, the lack of AQP-4 positive endfeet observed in the PFC of post mortem brains from MDD patients prompted us to hypothesize that either the endfeet of astrocytes were primarily morphologically affected, becoming only secondarily devoid of AQP-4 expression, or AQP-4 was dyslocalized, although astrocyte processes remained unaffected. Using a staining for GFAP both in brain slices and in our cell culture model, we showed that in fact HAB cells did not show the same morphological complexity in slices *ex vivo* and did not possess the same amount of processes *in vitro* as NAB cells did, thus suggesting that astrocytes from this animal model were primarily morphologically affected. Astrocyte endfeet are essential to maintain the integrity of the BBB (Zhou et al., 2008), but also to mediate the uptake of several substances from the bloodstream. We might speculate that their impairment may explain the slow onset of AD action, as far as the recovery of a properly functional BBB is first necessary before ADs can be efficiently transported into the brain parenchyma. Therefore, we examined how NAB and HAB astrocytes were reacting to the AD FLX in terms of process extension. To our surprise, however, FLX induced an increased number of astrocyte processes in both cells, suggesting that a lack of AQP-4 on the BVs might not be a primary cause of disease, but may only affect the response to drug treatments. To better understand this aspect, we used RNAi to downregulate AQP-4 and examined how its knockdown was affecting the response to FLX. We thereby showed that, although control HAB astrocytes were still able to protrude an increased number of astrocyte processes in response to drug administration, both NAB and HAB transfected with siRNA targeting the mRNA coding for AQP-4 were not only becoming unable to protrude new astrocyte processes in response to FLX treatment, but they were also losing pre-existing ones (in NAB cells). Thus, suggesting that AQP-4 might exert multiple roles: on the one hand, it is necessary to somehow maintain astrocyte processes in shape, maybe through actin- (Nicchia et al., 2008) or GFAP-mediated mechanisms, as far as both these proteins may be important regulators of structural changes; on the other hand, AQP-4 is necessary to mediate AD efficacy. The latter is actually in line with reports showing that the mouse knockout for aqp-4 cannot recover from the stress-induced depressive-like behavior after FLX treatment (Kong et al., 2009). Our results from the RNAi experiments actually suggest that the observed lack of response to FLX in aqp-4 knockouts may depend on a reduced number of processes in astrocytes due to AQP-4 deficiency, which consequently hinders a FLX-induced process extension necessary to recover a proper astrocytic functionality. Although interesting, our results can only provide limited evidence for either temporary or persistent FLX effects on astrocyte processes after the short-term treatment used here. To clarify this point, washout experiment in cell culture would be needed and, if resulting effects turned out to be stable enough, then cell culture of astrocytes from adult animals treated with FLX might be important to further confirm these *in vitro* findings.

Our *ex vivo* results after short term treatment of NAB and HAB animals with FLX did not show the rescue of coverage of BVs by AQP-4-IR astrocyte processes, probably due to a delay in the uptake of FLX *in vivo* or to an insufficient time to relocate AQP-4 to the endfeet, which might require longer treatment exposures or the supportive role of other cell types, such as endothelial cells, necessary to induce the localization of AQP-4 to endfeet (Camassa et al., 2015). In fact, endothelial cells would be the first cells exposed to FLX, strongly suggesting future studies to analyze their response to FLX and ADs in more details, specifically to dissect the potential contribution of endothelial cells to the polarized distribution of AQP-4 in response to drug administration. Indeed, such studies should help to understand the molecular motors which regulate AQP-4 distribution and function. A better understanding of the mechanism(s) which drive AQP-4 in its appropriate localization may be of help to identify novel target molecules which could fasten the restoration of coverage of BVs by endfeet of astrocytes enriched in AQP-4 to favor the recovery of a functional BBB and substance exchange between bloodstream and brain parenchyma.

Another possibility to explain the lack of rescue effect that we observed in HAB rats treated with FLX might rely on a difference in the type of processes that might form after drug administration. We indeed observed that HAB cells treated with FLX are enriched in processes which appeared to be shorter than those induced in NAB cells, suggesting that longer treatment times may be necessary to fully recover an amount of astrocyte processes with proper functionality, i.e., long enough to reach and completely surround again BVs with their AQP-4-IR endfeet. A similar study has been recently published, which showed that the AD naltrexone could reverse an astrocytic atrophy revealed in macaques with behavioral disorders (Lee et al., 2015).

Although Nicchia et al. (2008) have shown that stellation of astrocytes does not directly depend on the presence of AQP-4, we noticed that AQP-4 knockdown resulted in a lack of stellate morphology and FLX was no longer able to induce such a stellate phenotype in AQP-4 knockdown astrocytes. We cannot rule out that such a phenotype is due to a primary disrupted actin or GFAP cytoskeleton in HAB astrocytes, since we observed that GFAP is strongly impaired in HAB brains. More studies are needed to identify the specific molecular mechanisms behind the lack of stellate morphology in HAB cells.

## Conclusion

Our results show that AQP-4 might be necessary in order to maintain astrocyte processes, which are the functional unit of astrocytes at the BBB and around synapses (Kimelberg, 2010). Moreover, we could show that the lack of AQP-4 impairs the effect of FLX in restoring basal amounts of processes per cell. We think that a better understanding of the mechanisms which drive AQP-4 to the endfeet and around BVs might help to develop pharmacological compounds which could reverse disease phenotypes with an astrocytic pathology, such as MDD.

## Author Contributions

BDB conceived and designed the experiments for the work and contributed to acquisition, analysis, interpretation of data and to drafting of the manuscript; VAM, SB, LJ, and GG-G contributed to acquisition, analysis and interpretation of data for the work; IDN contributed to animal experiments (in particular to the generation and maintenance of the HAB rat colony) and to revision of the manuscript for important intellectual content; RR contributed to the conception of the work and to revision of the manuscript for important intellectual content. All authors revised and approved the final version of the manuscript to be published and agreed to be accountable for all aspects of the work.

## Conflict of Interest Statement

The authors declare that the research was conducted in the absence of any commercial or financial relationships that could be construed as a potential conflict of interest.

## Supplementary Material

**FIGURE S1 | The analysis of pixel intensity for AQP-4 staining in NAB **(A)** and HAB **(B)** cells revealed that both si2 and si3 showed a high degree of downregulation in both cell types, although with different efficiencies.** However, the reduced efficiency of si3 in HAB cells could also be explained through a general reduced amount of AQP-4 signal in HAB C cells with respect to the NAB C (NAB C, 49.01 signal intensity [in arbitrary unit] and HAB C, 36.48). ANOVA with Dunnett’s post hoc test, ^∗^*p* < 0.05, ^∗∗^*p* < 0.01.
