# Supplementary figures and images for: Fluoxetine Requires the Endfeet Protein Aquaporin-4 to Enhance Plasticity of Astrocyte Processes
Source: Front Cell Neurosci. 2016 Feb 2;10:8. doi: 10.3389/fncel.2016.00008 (PMC4735422; doi:10.3389/fncel.2016.00008)

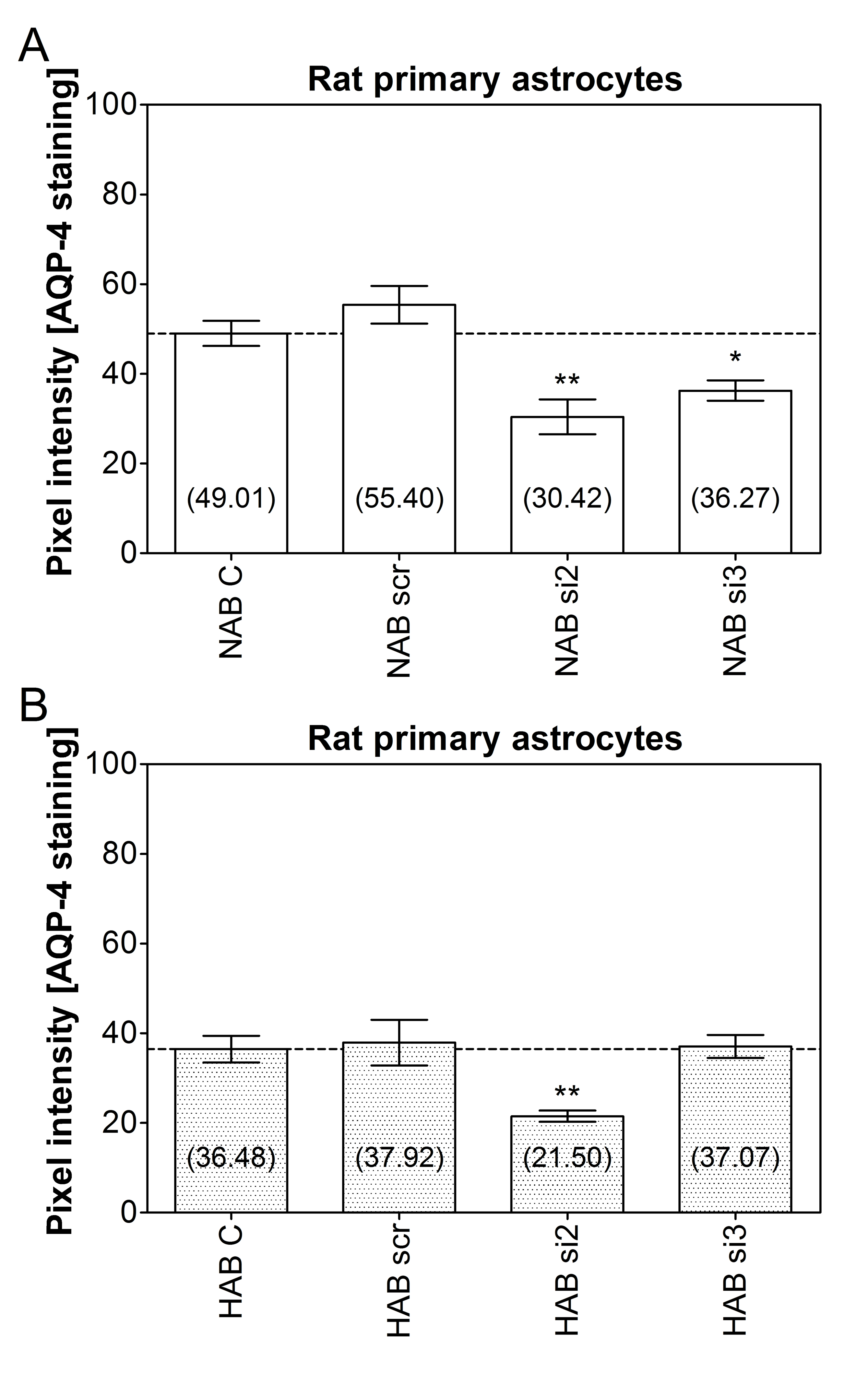

Supplement: Supplementary file 1 [file Image_1.TIF]
